# Reduction of nutrients, microbes, and personal care products in domestic wastewater by a benchtop electrocoagulation unit

**DOI:** 10.1038/srep09380

**Published:** 2015-03-23

**Authors:** E. M. Symonds, M. M. Cook, S. M. McQuaig, R. M. Ulrich, R. O. Schenck, J. O. Lukasik, E. S. Van Vleet, M. Breitbart

**Affiliations:** 1 University of South Florida, College of Marine Science, 140 7^th^ Avenue South, St. Petersburg, Florida, USA; 2 St. Petersburg College, 2465 Drew Street, Clearwater, Florida, USA; 3 BCS Laboratories, Inc., 4609-A NW 6^th^ Street, Gainesville, Florida, USA

## Abstract

To preserve environmental and human health, improved treatment processes are needed to reduce nutrients, microbes, and emerging chemical contaminants from domestic wastewater prior to discharge into the environment. Electrocoagulation (EC) treatment is increasingly used to treat industrial wastewater; however, this technology has not yet been thoroughly assessed for its potential to reduce concentrations of nutrients, a variety of microbial surrogates, and personal care products found in domestic wastewater. This investigation's objective was to determine the efficiency of a benchtop EC unit with aluminum sacrificial electrodes to reduce concentrations of the aforementioned biological and chemical pollutants from raw and tertiary-treated domestic wastewater. EC treatment resulted in significant reductions (p < 0.05, α = 0.05) in phosphate, all microbial surrogates, and several personal care products from raw and tertiary-treated domestic wastewater. When wastewater was augmented with microbial surrogates representing bacterial, viral, and protozoan pathogens to measure the extent of reduction, EC treatment resulted in up to 7-log_10_ reduction of microbial surrogates. Future pilot and full-scale investigations are needed to optimize EC treatment for the following: reducing nitrogen species, personal care products, and energy consumption; elucidating the mechanisms behind microbial reductions; and performing life cycle analyses to determine the appropriateness of implementation.

In order to protect public and environmental health, innovative technologies are needed to reduce the concentrations of emerging microbes^1^ and chemicals^2^ from domestic wastewater prior to discharge into the environment and/or water reuse. Fecal-borne pathogens, encompassing known and emerging bacteria, helminths, protozoa, and viruses, substantially contribute to human disease and mortality worldwide^1^,^3^. Furthermore, it has been postulated that the input of personal care products (PCPs; a chemically diverse group of over-the-counter medications, insect repellents, antibiotics, and disinfectants) into aquatic environments or the drinking water supply could negatively affect wildlife and humans, respectively^4^,^5^. Finally, it is well-understood that the removal of nutrients, principally nitrogen and phosphorus, from domestic wastewater is necessary to prevent the eutrophication of surface waters exposed to treated wastewater discharge. While many different wastewater treatment options exist, adequate reduction of all chemicals and microbes is extremely complex due to their great physical and structural diversity^1^,^6^. It is therefore important to evaluate treatment technologies for their ability to remove a diverse range of contaminants, since a combination of approaches will likely be required to ensure safe discharge of treated effluent and/or water reuse.

Electrocoagulation (EC) has become increasingly popular over the last 25 years to treat a wide-variety of wastewaters as technological advances have made this technique more cost- and energy-efficient^7^,^8^,^9^,^10^. The EC process applies electricity to sacrificial electrodes (typically aluminum or iron), which generates coagulants (e.g. aluminum hydroxide for an aluminum anode), destabilizes contaminants, enhances the suspension of particulates, and disrupts emulsions. Contaminants are either directly broken down or aggregated to form flocs that become buoyant as they associate with the gases generated by the concurrent electrolysis of water. Following EC, the floc is separated from the treated water via sedimentation and/or filtration. EC may be an advantageous treatment option as it does not require a constant supply of chemicals^7^,^8^,^9^,^10^ and consequently, may be more easily implemented in a developing-country context where such chemicals are not readily available^7^. It has also been suggested that EC technology could be an effective decentralized drinking water treatment technology^11^ and easily deployed as portable equipment for use in remote locations or in the event of emergencies^12^.

The efficacy of EC to reduce various biological and chemical constituents found in water and wastewater under normal and emergency conditions has been investigated in several prior studies. When evaluating EC technologies for their use in treating potable water, Vik et al. determined significant removal of humic substances with EC treatment of surface waters^13^ and Zhu et al. ascertained effective removal of MS2 bacteriophages from synthetic freshwater^14^. In another study, even though the use of iron electrodes in EC treatment reduced MS2 bacteriophage by up to 6.5-log_10_ in synthetic freshwater, natural levels of organic matter present in surface waters limited virus reductions to as little as 1.0-log_10_^15^. Consequently, the use of aluminum electrodes was suggested to prevent the complexation of organic matter and iron ions that inhibit adequate flocculation and subsequent virus removal. Furthermore, EC treatment of surface waters is both technically and economically effective for the removal of algae^16^ and greatly reduces concentrations of fecal indicator bacteria^17^. In a recent laboratory study, EC decreased concentrations of the antibiotic tetracycline by nearly 99% in laboratory-made aqueous solutions^18^. With respect to the treatment of industrial wastewater, EC has also been extensively used, primarily with aluminum, iron, and steel electrodes, to reduce chemical oxygen demand (COD) as well as the concentrations of arsenic, fluoride, food and oil waste, heavy metals, nitrate, organic matter, phenolic and polymeric wastes, suspended particles, textile dyes, and refractory organic pollutants^6^,^7^,^9^,^19^,^20^,^21^.

Unlike industrial wastewater, the application of EC to treat domestic wastewater has yet to be as thoroughly investigated. To date, several studies have shown that EC treatment of domestic wastewater (natural and synthetic) can greatly reduce turbidity by more than 90%, COD by up to 75%, and provide up to ‘complete disinfection' per the absence of fecal coliforms in treated effluents^17^,^22^,^23^,^24^,^25^,^26^,^27^,^28^. Additionally, Ozyonar et al. observed phosphorus removal efficiencies as great as 98% with EC treatment of domestic wastewater and determined that aluminum electrodes provided the greatest removal of phosphorus, as well as COD and turbidity^28^. The incorporation of EC as a tertiary or polishing treatment has also been suggested as it can greatly reduce phosphate concentrations in domestic wastewater after secondary treatment via anaerobic digestion or activated sludge treatment^23^,^29^.

The application of EC to treat domestic wastewater represents a potential alternative and/or addition to traditional treatment due to cost effectiveness, ease in operation, design simplicity, and its successful use to treat other waters; however, the full potential of EC to reduce multiple types of microbes, PCPs, and nitrogen species from domestic wastewater has yet to be fully understood^7^,^30^. The primary objective of this study was to determine the efficiency of a benchtop EC unit, equipped with aluminum electrodes, to reduce nutrients (nitrate + nitrite, nitrite, ammonium, and phosphate), a suite of 18 PCPs, and six types of microbes from both raw and tertiary-treated domestic wastewater in order to further understand the potential of EC as a principal or polishing treatment, respectively. The PCPs assessed in this study represent those identified as potential threats to environmental and/or human health and routinely studied in U.S. Environmental Protection Agency (US EPA) Clean Water Act programs^31^. To investigate the potential of EC treatment to reduce microbial contaminants, several commonly used microbial surrogates were chosen to represent bacterial, parasitic protozoan and viral pathogens as well as to allow for culture- and molecular-based analyses.

## Results and discussion

### Nutrients

Significant (>95%; p < 0.0003, α = 0.05) reductions in phosphate were observed upon EC treatment of both raw wastewater and tertiary treated wastewater (Table 1). These results corroborate previous findings demonstrating up to 100% removal using aluminum sacrificial electrodes and further suggest that EC may be an especially useful treatment technology to achieve enhanced phosphorus reductions from domestic wastewater^28^,^29^,^32^. Despite the consistent reduction of phosphate by EC, the extent of reduction for the other nutrients differed for raw wastewater compared to tertiary-treated wastewater (Table 1).

Significant decreases in nitrate + nitrite were observed (48.35%; p = 0.0007, α = 0.05) during the treatment of tertiary-treated wastewater; however, no significant reduction in nitrate + nitrite was achieved during the treatment of raw wastewater. Additionally, even though significant increases in nitrite and ammonium were observed during EC treatment of tertiary-treated wastewater, significant reductions (>14%; p < 0.0087; α = 0.05) were observed after EC treatment of raw wastewater. Previous studies on nitrate reduction from ground and surface water for potable water treatment have shown that EC with iron and aluminum blades is more efficient than chemical coagulation; however, the extent of nitrate reduction depended upon the EC conditions (e.g. current density applied, electrode connections) and the characteristics of the water under treatment (e.g. pH, initial nitrate concentration, total dissolved solids)^33^,^34^. Since up to 89.7% nitrate removal from aqueous solutions has been observed by Malakootian et al.^34^, future research is necessary to identify the optimum EC conditions for reduction of various nitrogen species from domestic wastewater and treated effluent.

### Microbes

Six commonly used microbial surrogates were analysed using a combination of molecular- and culture-based techniques. The double-stranded DNA human polyomavirus (HPyV) and single-stranded RNA pepper mild mottle virus (PMMoV) were measured as surrogates for DNA and RNA viruses in wastewater, respectively, using molecular techniques^35^,^36^,^37^,^38^. Fecal-indicator bacteria (FIB; fecal coliforms and *Enterococcus* spp.) were measured as surrogates for wastewater-related bacteria using culture-based techniques as well as molecular techniques for *Enterococcus* spp.^3^,^39^. To quantify the extent of microbial reduction, the EC unit was used to treat domestic wastewater augmented with the aforementioned bacteria and viruses as well as two other commonly used microbial surrogates that are not typically found in wastewater at high concentrations: male-specific (F+) bacteriophages (MS2)^40^ and *Bacillus subtilis* spores (surrogate for wastewater-related, protozoan parasites; i.e *Cryptosporidium*^3^). Both MS2 bacteriophages and *B. subtilis* spores were analysed using culture-based techniques.

EC treatment resulted in significant reductions (p < 0.0286, α = 0.05), ranging from 81.567% to >99.999998%, of all microbial surrogates tested in all domestic wastewater samples (Table 2). These results suggest that EC with aluminum electrodes is an effective treatment for the wide-range of pathogen types present in domestic wastewater. Furthermore, EC treatment resulted in a greater than 4-log_10_ reduction for all microbial surrogates in augmented domestic wastewater. Although this study does not attempt to discern the mechanisms behind “the observed reductions after” EC treatment, previous studies on synthetic freshwater and wastewater have suggested that the primary microbial removal mechanism during EC is due to the enmeshment of microbes to flocs and subsequent separation of flocs from treated water by filtration^12^. It is also possible that the oxidants produced during EC (e.g. HO·, O_3_, H_2_O_2_) provide additional microbial reductions via disinfection as a result of cell/capsid membrane damage^15^,^17^. The effective reduction of FIB observed (as great as 7-log_10_) in this study supports the results of previous investigations on EC treatment of domestic wastewater, which cite reductions as high as 4-log_10_^17^,^22^,^23^,^25^. Finally, this is the first study to our knowledge to demonstrate that EC can significantly reduce concentrations of viral and parasitic protozoan surrogates in domestic wastewater.

### Personal care products

EC treatment of raw domestic wastewater significantly (p < 0.05, α = 0.05) reduced concentrations of the following PCPs: acetaminophen, DEET, gemfibrozil, ibuprofen, iopromide, salicylic acid, triclocarban, and triclosan (Table 3). While the initial concentrations of many PCPs in tertiary-treated wastewater were below the process limit of detection (pLOD), EC treatment of tertiary-treated wastewater significantly (p < 0.05, α = 0.05) decreased concentrations of iopromide, sulfamethoxazole, and thiabendazole (Table 3). Although this study does not attempt to discern the EC removal mechanisms associated with the different PCPs, it is likely that PCP adsorption to flocs was a major removal mechanism^41^, particularly for compounds with higher octanol-water partition coefficient (K_ow_) values (e.g. gemfibrozil, ibuprofen, triclocarban, and triclosan). It is also possible that compounds with lower K_ow_ values (e.g., acetaminophen, DEET, iopromide, salicylic acid, sulfamethoxazole, and thiabendazole) were removed by the destabilizing effects of EC, which result in charge neutralization, decreased solubility, and ultimately, enhanced aggregation to flocs^42^.

The differences in PCP removal by EC treatment observed for raw wastewater and tertiary-treated wastewater are likely the result of chemical differences between the two water types (e.g. total suspended solids, which differed on average by two orders of magnitude that influence chemical adsorption to flocs^7^,^8^,^9^,^10^,^41^,^42^ (195 mg/L and 1 mg/L in raw wastewater and tertiary-treated wastewater, respectively; courtesy of South Cross Water Reclamation Facility)). Since it has been previously reported that current intensity greatly influences the extent of tetracycline (a common antibiotic) removal from aqueous solutions during EC with aluminum electrodes^18^, it is possible that the current intensity was suboptimal for maximizing PCP removal during this study. Future research is need to optimize the EC treatment process for removal of a wide-range of PCPs from domestic wastewater after various primary and secondary treatments in order to understand the full potential of EC to reduce PCP concentrations.

## Conclusions

This study demonstrates that a benchtop EC unit outfitted with aluminum electrodes can concomitantly reduce concentrations of phosphate, microbial surrogates representing several major pathogen types (DNA/RNA viral, bacterial, protozoan parasite), as well as several PCPs in domestic wastewater. By providing the first information about the ability of EC to reduce concentrations of viral and parasitic protozoan surrogates, as well as PCPs, this study enhances previous assertions that EC is a promising sustainable wastewater treatment technology for domestic wastewater^7^,^12^,^30^. While these collective results highlight the potential of EC for domestic wastewater treatment, further research is needed to address a number of outstanding issues. First, future work should attempt to discern the mechanisms behind the observed reductions as well as to optimize EC configurations and conditions to enhance the removal of PCPs and nitrogen species from domestic wastewater. Secondly, it will be necessary to optimize the EC treatment conditions to minimize energy consumption and the incorporation of renewable energy sources^43^. Future pilot-scale and full-scale studies assessing the effectiveness of EC treatment of domestic wastewater are needed to fully understand the feasibility of this treatment option with respect to removing nutrients, microbes, and PCPs both from raw wastewater as a stand-alone treatment or as a polishing technology for refining tertiary-treated wastewater from standard wastewater treatment plants. Additionally, full life-cycle assessments are needed in order to understand the appropriateness of EC technologies as an option for decentralized and/or centralized domestic wastewater treatment prior to their implementation.

## Methods

### Benchtop electrocoagulation unit

**Figure 1:**
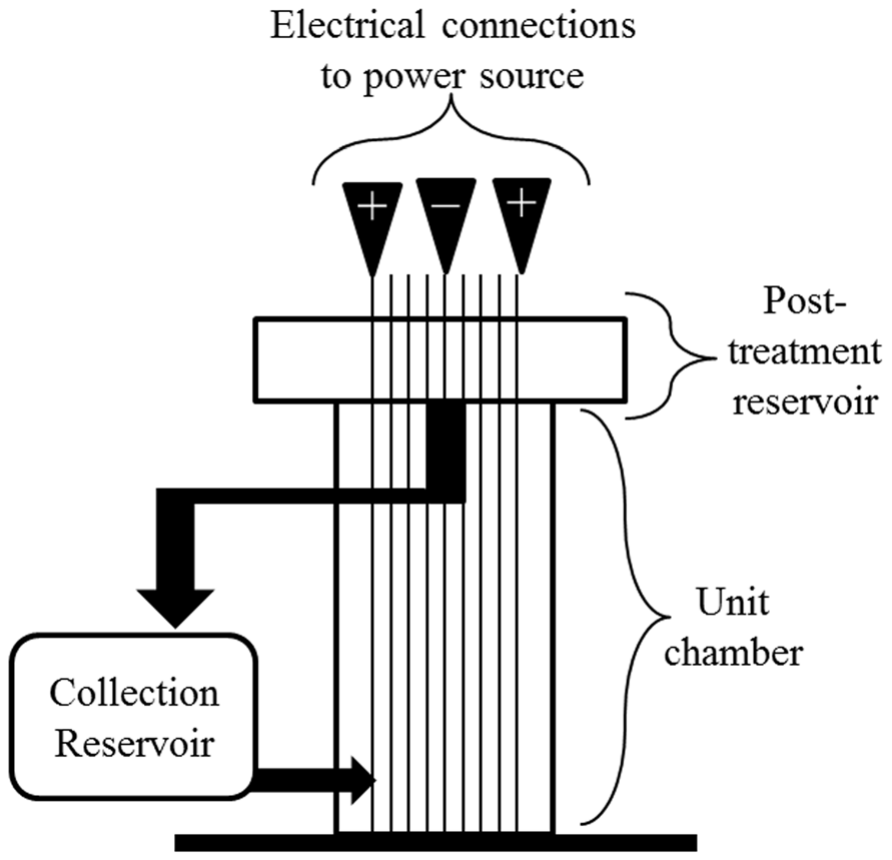
The benchtop electrocoagulation unit with nine aluminum blades arranged vertically in the unit chamber. Electrical connections on the first, fifth, and ninth blades were connected to a 110-volt AC to DC power converter. A peristaltic pump re-circulated wastewater up through the unit chamber, into the post-treatment reservoir, and into the collection reservoir.

The demonstration, benchtop EC unit (United States patent number 7211185 B2 by Powell Water Systems, Inc.; Centennial, CO, USA) evaluated in this study was comprised of a non-conductive, acrylic-resin chamber (35.6 × 5.4 × 2.5 cm) with nine aluminum plates (each 36.8 × 2.5 × 0.3 cm) vertically arranged and spaced 0.3 cm apart such that they occupied approximately 45% of the chamber volume (Figure 1). A 110-volt AC to DC power converter, set to 98 volts, was used to supply electricity to the unit via three electrical connections to the first, fifth, and ninth blade, resulting in two anodes and one cathode. During EC treatment, the actual current delivered ranged from 8.5–15.0 amps for raw domestic wastewater and 12.0–15.5 amps for tertiary-treated domestic wastewater. A peristaltic pump (Cole-Parmer® Masterflex Peristaltic Pump System 77910; Vernon Hills, IL, USA) was used to pump wastewater up through the unit chamber, which recirculated wastewater throughout the benchtop unit at a rate of 0.94 L/min. Wastewater was recirculated for 1 min per every liter of wastewater being treated. The resulting flocculant was removed from the EC unit effluent via filtration with paper filters that retain 11 μm particles (Whatman Qualitative Grade Plain Circles Grade 1; GE Healthcare Bio-Sciences, Pittsburgh, PA, USA). Since the aluminum blades become oxidized over time, they were cleaned with a sandblaster after every 12 L of wastewater treated by the EC unit to physically remove the oxidized portion of the aluminum blade.

### Experimental design

Raw influent (post-grit removal) and tertiary-treated (de-chlorinated) effluent were collected in sterile, plastic HDPE carboys from South Cross Bayou Water Reclamation facility (activated sludge plant with tertiary treatment) in St. Petersburg, Florida, USA. The tertiary-treated domestic wastewater received the following treatment prior to collection: grit removal, primary clarification, secondary treatment with an activated sludge system, and finally tertiary treatment with sand filtration, chlorination, and de-chlorination. Carboys were stored at 4°C in the dark and all experiments were conducted within 12 h of collection. Given the large number of analytes and logistical limitations, twice the minimum anticipated number of trials (n = 4) were collected before and after EC treatment in order to test the reduction efficiency of the EC unit. Four trials were executed with both raw wastewater and treated effluent, with each trial requiring an 18-L sample. From each sample, 6.1 L were isolated before treatment and the remaining volume was treated with the EC unit and filtered as described above. The EC unit was cleaned with 1 L analytical grade methanol and rinsed with 5 L DI water after each trial. Process controls, consisting of DI water that was recirculated through the EC unit, were collected after the second and fourth trial to ensure no cross-contamination between trials. All pre- and post-treatment samples, as well as process controls, were analyzed for nutrients, microbes, and PCPs.

In order to quantify the reduction efficiency of microbes, 1-L wastewater influent and effluent samples were augmented separately with concentrated surrogates for bacteria (*Enterococcus faecalis* ATCC-29212™ and *Escherichia coli* strain C600), viruses (JC HPyV ATCC-VR-1583™, PMMoV (obtained from Scott Adkins; USDA), and MS2 bacteriophages), and parasitic protozoa (*B. subtilis* spores) (see Supplementary Information). Four trials were executed for both the raw wastewater and tertiary-treated effluent. Twenty-milliliter and 120-ml aliquots of the spiked-wastewater were collected prior to treatment with the EC unit for samples augmented with bacteria and viruses, respectively. The remaining volume was treated with the benchtop EC unit as described above. The EC unit was cleaned between each trial and one process control was collected upon completion of the fourth trial.

### Nutrient analyses

Four sets of pre- and post-EC treatment samples of raw wastewater and tertiary-treated wastewater samples, along with two process controls, were analyzed in duplicate by the Oceanic Nutrient Laboratory at the University of South Florida, College of Marine Science for nitrate + nitrite, nitrite, ammonium, and phosphate. Due to the high nutrient concentrations in raw wastewater, pre-EC treatment raw wastewater samples were diluted to 2.4% final concentration with deionized water prior to analysis. The analytical methods used for nitrate + nitrite, nitrite, ammonium, and phosphate followed the recommendations of Ref. 44 and were analyzed using a five-channel Technicon Autoanalyzer II (SEAL Analytical, Mequon, WI, USA) upgraded with new heating baths, proportional pumps, colorimeters, improved optics, and an analog to digital conversion system (New Analyzer Program v. 2.40; Waters Corporation, Milford, MA, USA). To extend the dynamic range to 30 μM, the ammonium technique was modified by decreasing the flow rates for the nitroprusside, hypochlorite, phenolate, citrate, sample, air bubble, and waste draw to 50 μl, 50 μl, 50 μl, 320 μl, 600 μl, 160 μl, and 1200 μl per minute, respectively.

Nutrient standards were run in triplicate before and after analysis, as well as a check standard in the middle of the run to correct for any drift in sensitivity. The detection limits for nitrate + nitrite, nitrite, ammonium, and phosphate were 0.22 μM, 0.02 μM, 0.38 μM, and 0.09 μM, respectively. All method blanks were negative. Process controls for both experiments had low levels of nitrate + nitrite, nitrite, ammonium, and phosphate; however, the concentrations were less than the standard deviations for replicate samples.

### Microbial analyses

#### Human polyomavirus (HPyV) and pepper mild mottle virus (PMMoV)

All samples were processed as previously described^45^. Briefly, 12 ml of sample were 0.45-μm filtered and concentrated to 200 μl using Amicon Ultra-15 centrifugal filter units (EMD Millipore, Billerica, MA, USA). Viral concentrates were stored at 4°C overnight and DNA and RNA were simultaneously purified within 24 h of the experiment using the QIAmp MinElute Virus Spin Kit (Qiagen, Valencia, CA, USA), following manufacturer's instructions and eluting with 50 µl molecular grade water. cDNA was immediately generated by reverse transcription from RNA templates using random hexamers and Superscript III First Strand Synthesis for RT-PCR (Invitrogen, Carlsbad, CA, USA) per manufacturer's instructions. Extraction blanks, containing only the kit reagents, were also processed to ensure no cross-contamination among samples. RNA was stored at −80°C and DNA and cDNA were stored at −20°C.

Using previously published assays, quantitative PCR (qPCR) was used to determine the concentrations of HPyV^36^ and PMMoV^35^ following the established guidelines for qPCR^46^ (see Supplementary Information). The lowest standard dilution within the linear dynamic range of the standard curve was considered the limit of quantification (LOQ) and was 500 and 100 target gene copies per reaction for HPyV and PMMoV, respectively. When no fluorescence was detected, the concentration of HPyV and PMMoV was considered ‘less than the limit of detection' (<LOD). If fluorescence was detected at levels less than the LOQ, then the concentration was reported as positive but below the LOQ (+BLOQ). All extraction blanks and no-template controls were negative and PCR inhibition was only observed in one process control for the HPyV assay. All process blanks were negative for HPyV. For PMMoV, the process blanks were all negative except those that were +BLOQ for the experiments executed with augmented wastewater. Mean virus-target concentrations were back-calculated to reflect all sample dilutions (nucleic acid purification through qPCR detection) and the original sample volume concentrated. The process limit of quantification (pLOQ), an ideal estimation assuming 100% recovery, was 417 targets/ml for HPyV qPCR and 219 targets/ml for PMMoV RT-qPCR. The process limit of detection (pLOD) was assumed to be half the pLOQ for both assays.

#### Fecal indicator bacteria

To determine the concentrations of fecal indicator bacteria (FIB) in all natural and augmented domestic wastewater samples before and after EC treatment as well as in all process controls, multiple dilutions of each sample were filtered onto gridded, 0.45-µm-pore size nitrocellulose filters (Millipore, Billerica, MA, USA) in triplicate. Fecal coliforms were cultured on mFC agar^47^, with incubation at 44 ± 0.5°C for 24 h. All blue colonies were considered fecal coliforms and used to enumerate total concentrations of fecal coliforms. Enterococci were enumerated on mEI agar, with incubation at 41 ± 0.5°C for 48 h^39^. Resulting bacterial colonies with a blue halo were recorded as enterococci. The maximum volume filtered was 100 ml; therefore, the theoretical process limit of detection (pLOD) was 1 colony forming unit (cfu)/100 ml. No FIB colonies grew on method blanks or process controls.

The concentration of enterococci in the augmented domestic wastewater samples was also determined using qPCR following standard methods^48^ and internal control nucleic acid based sequence amplification (IC-NASBA) (see Supplementary Information). Briefly, 1 ml volumes of augmented domestic wastewater before and after EC treatment were filtered onto 0.45-µm-pore size HV polyvinylidene difluoride filters (Millipore, Billerica, MA, USA) within 24 h of collection. RNA was purified from filters designated for IC-NASBA analysis using the RNeasy® Mini Kit (Qiagen, Valencia, CA, USA). DNA was purified from filters designated for enterococci qPCR using the DNeasy® Blood & Tissue Kit (Qiagen, Valencia, CA, USA). RNA and DNA were eluted using 50 μl and 100 μl of nuclease-free water, respectively. All samples were analyzed in triplicate. The LOQ for the qPCR and IC-NASBA assays was 100 cells per reaction and results were reported as +BLOQ or <LOD, as previously described for HPyV and PMMoV. The pLOQ was 5,000 cells/ml for the qPCR analysis and 2,000 cells/ml for the IC-NASBA analysis. The pLOD was assumed to be half the pLOQ for both assays. All extraction blanks and no-template controls were negative and no PCR inhibition was observed. All process blanks were negative.

#### Bacillus subtilis spores

All augmented pre-and post-EC treatment samples and process controls were incubated at 50°C for 20 min to kill other non-spore forming bacteria and then maintained in the dark at 4°C. Within 48 h of the experiment, aliquots of each sample were spread-plated in triplicate (all pre-EC treatment samples were diluted 1:10,000) onto tryptic soy agar and incubated at 36.5 ± 1°C for 24 h. The resulting viable *B. subtilis* colonies (i.e. opaque in color and rough appearance) were enumerated and concentrations were back-calculated to account for dilutions. Since the maximum sample volume plated was 500 μl, the pLOD was 2 cfu/ml. No colonies grew on method blanks. While no colonies were present in the process control for the experiments with raw wastewater, the average *B. subtilis* concentration in the process control for the experiment with the tertiary treated wastewater was 39 cfu/ml.

#### MS2 bacteriophage

Since the wastewater samples were augmented with an MS2 bacteriophage culture prior to EC treatment, MS2 bacteriophage concentrations were quantified using the single-agar layer (SAL) protocol using *E.coli* Famp ATCC-700891^TM^ for post-EC treatment samples and the double-agar layer (DAL) protocol for pre-EC treatment samples that had been diluted four-fold^40^. Per US EPA method 1602, each pre-EC treatment sample was analyzed using the DAL protocol in triplicate and each post-EC treatment sample was analyzed in replicates of ten using the SAL protocol. All method blanks were negative. The pLOD was 1 plaque forming unit (pfu)/10 ml for the SAL protocol and 2,000 pfu/ml for the DAL protocol. The average concentrations of MS2 bacteriophage in the process controls were less than the pLOD.

### Personal care products

Four raw and four tertiary treated wastewater samples as well as two process controls were analyzed before and after EC treatment by Test America (a NELAP accredited laboratory; Sacramento, CA, USA) following US EPA method 1694 for the following PCPs (with pLOD for all samples except the raw wastewater prior to EC treatment indicated in parentheses): acetaminophen (20 ng/L), caffeine (51 ng/L), carbamazepine (10 ng/L), DEET (25 ng/L), gemfibrozil (25 ng/L), primidone (250 ng/L), salicyclic acid (50 ng/L), thiabendazole (10 ng/L), triclocarban (10 ng/L), triclosan (50 ng/L), warfarin (20 ng/L), ibuprofen (25 ng/L), iopromide (50 ng/L), meprobamate (10 ng/L), naproxen (50 ng/L), phenytoin (100 ng/L), sulfamethoxazole (10 ng/L), and trimethoprim (10 ng/L)^31^. For the analysis of raw wastewater prior to EC treatment, the LOD was an order of magnitude greater for all analytes.

No PCPs were detected in the two process controls collected during the experiment with tertiary-treated wastewater. However, low concentrations of acetaminophen (22 ng/L), caffeine (83 ng/L), DEET (180 ng/L), and salicylic acid (76 ng/L) were detected in the process controls collected during the experiment with raw wastewater. Since the detected concentrations of these analytes in the process controls are less than the standard deviations observed for raw wastewater samples before and after EC treatment, it is unlikely that the observed contamination influenced the results of this study.

### Statistical analyses

Statistical analyses were executed in SAS v.9.3 (SAS Institute Inc.; Cary, NC, USA) to identify significant (α = 0.05) differences in the concentrations of all nutrients, microbes, and PCPs before and after EC treatment. If the data had normal distributions, a two-tailed t-test was performed with either the pooled method (for equal variances) or the Satterthwaite approximation (for unequal variances). If the data were not normally distributed, then the non-parametric Wilcoxon Rank Sum test was performed. For a given analyte, if a significant difference in pre- and post-EC treatment concentrations was determined with 95% confidence, then the average percent reduction was calculated. If concentrations were +BLOQ or <LOD, then the pLOQ or pLOD, respectively, were used to conservatively test for statistical differences and to calculate the average percent reduction.

## Author Contributions

This study was designed by E.M.S., M.M.C., E.S.V.V. and M.B., with assistance from J.O.L. E.M.S., M.M.C., S.M.M., R.M.U. and R.O.S. executed the experiments with input from J.O.L. Microbial analyses were executed by R.O.S. (MS2 bacteriophage), S.M.M. (fecal indicator bacteria, *B. subtilis* spores, and HPyV), R.M.U. (fecal indicator bacteria), and E.M.S. (*B. subtilis* spores and PMMoV). The *B. subtilis* spores were provided by J.O.L. Statistical analyses were executed by E.M.S., with assistance from M.M.C. The manuscript text as well as tables and figures were written and prepared by E.M.S., with subject relevant contributions from all authors. All authors reviewed the manuscript.

## Supplementary Material

Supplementary Information Supplementary Information

## Figures and Tables

**Table 1:** Mean +/− standard deviation of nutrient concentrations before and after EC treatment of raw wastewater and tertiary-treated wastewater with the benchtop unit. A negative t-statistic signifies an increase in nutrient concentrations after EC treatment. When a two-tailed student's t-test (t) or Wilcoxon Rank Sum test (S) revealed a positive, significant difference (α = 0.05) between pre- and post- EC treatment concentrations, the mean percent reduction was calculated

		Mean concentration +/− standard deviation (μM)			
Wastewater Sample	Nutrient	Pre-EC treatment	Post-EC treatment	Two-tailed student's t-test results	% Mean reduction
Raw	Nitrate + Nitrite	11.20 +/− 0.79	10.63 +/− 0.31	t = 1.36, p = 0.2218	N/A
	Nitrite	0.52 +/− 0.07	0.33 +/− 0.06	t = 3.83, p = 0.0087	64.48
	Ammonium	1349.34 +/− 10.88	1155.76 +/− 63.67	t = 5.99, p = 0.0079	14.35
	Phosphate	62.76 +/− 20.17	2.65 +/− 0.42	t = 19.93, p = 0.0003	95.79
Tertiary-treated	Nitrate + Nitrite	2.88 +/− 0.22	1.49 +/− 0.38	t = 6.34, p = 0.0007	48.35
	Nitrite	0.11 +/− 0.03	0.41 +/− 0.02	t = −15.87, p < 0.0001	N/A
	Ammonium	3.08 +/− 0.19	4.78 +/− 0.29	t = −9.74, p < 0.0001	N/A
	Phosphate	3.95 +/− 0.20	0.15 +/− 0.02	t = 37.87, p < 0.0001	96.33

**Table 2:** Mean +/− standard deviation (n = 4 unless indicated otherwise; *^a^* n = 2 and *^b^* n = 3) of bacteria and virus concentrations, two-tailed student's t-test (t) or Wilcoxon Rank Sum test (S) results, and mean percent reduction from domestic wastewater before and after EC treatment. Analyte concentrations are described as less than the process limit of detection (<pLOD) when undetected: *^c^*0.01 fecal indicator bacteria cfu/ml, *^d^*2.08 × 10^2 ^HPyV targets/ml, *^e^*1.09 × 10^2^ PMMoV targets/ml, *^f^*0.1 MS2 bacteriophage pfu/ml, and *^g^*1.00 × 10^3 ^*Enterococcus* spp. targets/ml with IC-NASBA. For molecular analyses, analyte concentrations are considered positive but below the process limit of quantification (+BLOQ) when at least one replicate is +BLOQ: *^h^*5.00 × 10^3 ^*Enterococcus* spp. targets/ml (qPCR), *^i^*2.00 × 10^3^ *Enterococcus* spp.targets/ml (IC-NASBA), and *^l^*2.19 × 10^2^ PMMoV targets/ml

		Mean concentration +/− standard deviation per milliliter					
Wastewater Sample	Analyte	Pre-EC treatment	Post-EC treatment	Test statistic and p-value		% Mean reduction	
Raw	*Enterococcus* spp. (cfu)	4.701 × 10^2^ +/− 1.50 × 10^2^	<pLOD*^c^*	S = 26.00, p = 0.0286		>99.998	
	Fecal coliform (cfu)	1.95 × 10^2^ +/− 2.29 × 10^2^	< pLOD*^c^*	S = 26.00, p = 0.0286		>99.995	
	HPyVs (qPCR target)	2.69 × 10^3 ^+/− 8.16 × 10^2^	< pLOD*^d^*	S = 26.00, p = 0.0286		>92.252	
	PMMoV (qPCR target)	5.47 × 10^4^ +/− 1.64 × 10^4^	<pLOD*^e^*	S = 26.00, p = 0.0286		>99.800	
Tertiary- treated	*Enterococcus* spp. (cfu)	5.43 × 10^−2^ +/− 2.54 × 10^−2^	<pLOD*^c^*	S = 26.00, p = 0.0286		>81.567	
	Fecal coliform (cfu)	7.40 × 10^1^ +/− 4.05 × 10^1^	< pLOD*^c^*	S = 26.00, p = 0.0286		>99.986	
	HPyVs (qPCR target)	<pLOD*^d^*	< pLOD*^d^*	N/A		N/A	
	PMMoV (qPCR target)	<pLOD*^e^*	<pLOD*^e^*	N/A		N/A	
Spiked raw	*Bacillus subtilis* (cfu)	3.80 × 10^6^ +/− 3.74 × 10^5^	3.00 × 10^0^ +/− 1.41 × 10^0^ *^a^*	S = 26.00, p = 0.0286		>99.99996	
	*Enterococcus* spp. (cfu)	1.25 × 10^8^ +/− 1.56 × 10^7^	2.15 × 10^0^ +/− 4.12 × 10^0^	S = 26.00, p = 0.0286		>99.999998	
	*Enterococcus* spp. (qPCR target)	1.37 × 10^6^ +/− 1.40 × 10^5^	+BPLOQ*^h^*	S = 26.00, p = 0.0286		>99.507	
	*Enterococcus* spp. (NASBA target)	1.15 × 10^6^ +/− 1.72 × 10^5^	<pLOD*^g^*	S = 26.00, p = 0.0286		>99.913	
	Fecal coliform (cfu)	1.22 × 10^6^ +/− 2.61 × 10^5^	8.13 × 10^−1^ +/− 9.10 × 10^−1^ *^b^*	t = 9.33, p = 0.0026		>99.99993	
	HPyVs (qPCR target)	6.29 × 10^5^ +/− 2.00 × 10^5^	< pLOD*^d^*	S = 26.00, p = 0.0286		>99.967	
	PMMoV (qPCR target)	6.38 × 10^6^ +/− 2.35 × 10^6^	<pLOD*^e^*	S = 26.00, p = 0.0286		>99.998	
	MS2 bacteriophage (pfu)	3.72 × 10^4^ +/− 3.82 × 10^3^	<pLOD*^f^*	S = 26.00, p = 0.0286		>99.9997	
Spiked tertiary- treated	*Bacillus subtilis* (cfu)	3.60 × 10^6^ +/− 3.69 × 10^5^	7.45 × 10^2^ +/− 6.43 × 10^2 *a*^	t = 20.23, p = 0.0003		>99.989	
	*Enterococcus* spp. (cfu)	1.44 × 10^6^ +/− 1.03 × 10^5^	5.67 × 10^1^ +/− 7.03 × 10^1^	t = 27.88, p = 0.0001		>99.996	
	*Enterococcus* spp. (qPCR target)	1.68 × 10^6^ +/− 1.40 × 10^5^	+BPLOQ*^h^*	S = 26.00, p = 0.0286		>99.702	
	*Enterococcus* spp. (NASBA target)	1.56 × 10^6^ +/− 1.04 × 10^5^	+BPLOQ*^i^*	S = 26.00, p = 0.0286		>99.872	
	Fecal coliform (cfu)	1.47 × 10^6^ +/− 6.24 × 10^4^	3.38 × 10^1^ +/− 4.59 × 10^1^	t = 47.05, p < 0.0001		>99.998	
	HPyVs (qPCR target)	7.69 × 10^5^ +/− 2.81 × 10^5^	< pLOD*^d^*	S = 26.00, p = 0.0286		>99.973	
	PMMoV (qPCR target)	1.24 × 10^7 ^+/− 6.26 × 10^6^	+BPLOQ*^l^*	S = 26.00, p = 0.0286		>99.998	
	MS2 bacteriophage (pfu)	2.98 × 10^4^ +/− 5.34 × 10^3^	<pLOD*^f^*	S = 26.00, p = 0.0286		>99.9996	

**Table 3:** Mean +/− standard deviation of personal care product concentrations before and after EC treatment of raw and tertiary-treated wastewater with the benchtop unit (n = 4, unless otherwise noted). Any undetected analytes are listed as less than the reported process limit of detection (<pLOD). When a two-tailed student's t-test (t) or Wilcoxon Rank Sum test (S) revealed a positive, significant difference (α = 0.05) between pre- and post- EC treatment concentrations, the mean percent reduction was calculated. Results that exceeded the calibration range but did not saturate the instrument detector are indicated with an ^E^. Results that were likely underestimations (laboratory control sample spike below the control limit) are indicated with*

	Raw wastewater				Tertiary-treated wastewater			
	Mean concentration +/− standard deviation (ng/L)				Mean concentration +/− standard deviation (ng/L)			
Analyte	Pre-EC treatment	Post-EC treatmet	Test statistic and p-value	% Mean reduction	Pre-EC treatment	Post-EC treatment	Test statistic and p-value	% Mean reduction
Acetaminophen	55750 +/− 28000^E^	28000 +/− 4243^E^	S = 26.00, p = 0.0286	49.78	<pLOD	<pLOD	N/A	N/A
Caffeine	31250 +/− 3202^E^	26250 +/− 4787^E^	S = 23.00, p = 0.1714	N/A	<pLOD	72 +/− 3	N/A	N/A
Carbamazepine	130 +/− 16	125 +/− 6	S = 20.00, p = 0.6564	N/A	165 +/− 6	178 +/− 10	N/A	N/A
DEET	4075 +/− 263	3400 +/− 424^E^	t = 2.70, p = 0.0354	16.56	29 +/− 1	68 +/− 12	N/A	N/A
Gemfibrozil	2700 +/− 13	2350 +/− 82^E^	t = 4.58, p = 0.0038	12.96	<pLOD	<pLOD	N/A	N/A
Ibuprofen	12000 +/− 817^E^	9950 +/− 900^E^	t = 3.37, p = 0.0150	17.08	<pLOD	<pLOD	N/A	N/A
Iopromide	4600 +/− 653*	3325 +/− 619*^, E^	S = 26.00, p = 0.0286	24.77	505 +/− 31	350 +/− 26	t = 7.67, p = 0.0003	31.00
Meprobamate	655 +/− 48	660 +/− 82^E^	t = 0.11, p = 0.9193	N/A	588 +/− 24^E^	645 +/− 44^E^	N/A	N/A
Naproxen	11250 +/− 500^E^	11250 +/− 500^E^	S = 18.00, p = 1.0000	N/A	<pLOD	<pLOD	N/A	N/A
Phenytoin	<pLOD	130 +/− 0 (n = 2)	N/A	N/A	183 +/− 15	208 +/−15	N/A	N/A
Primidone	<pLOD	<pLOD	N/A	N/A	<pLOD*	<pLOD*	N/A	N/A
Salicylic Acid	32500 +/− 5568	9425 +/− 961^E^	S = 26.00, p = 0.0286	71.00	61 (n = 1)	78 +/−15	N/A	N/A
Sulfamethoxazole	1925 +/− 310	1700 +/− 548^E^	t = 0.72, p = 0.5013	N/A	24 +/− 5	< RL	S = 26.00, p = 0.0286	58.76
Thiabendazole	<pLOD	15 +/− 1	N/A	N/A	20 +/− 1	18 +/− 1	t = 3.00, p = 0.0240	10.00
Triclocarban	728 +/−200	113 +/− 75	t = 5.77, p = 0.0012	84.54	<pLOD	<pLOD	N/A	N/A
Triclosan	1350 +/−238	248 +/− 89	t = 8.68, p = 0.0001	81.67	<pLOD	<pLOD	N/A	N/A
Trimethoprim	488 +/− 54	508 +/− 48^E^	t = 0.56, p = 0.5986	N/A	<pLOD	<pLOD	N/A	N/A
Warfarin	<pLOD	<pLOD	N/A	N/A	<pLOD	<pLOD	N/A	N/A
