# Supplementary material for: Reduction of nutrients, microbes, and personal care products in domestic wastewater by a benchtop electrocoagulation unit
Source: Sci Rep. 2015 Mar 23;5:9380. doi: 10.1038/srep09380 (PMC4369739; doi:10.1038/srep09380)
Supplement: Supplementary Information [file srep09380-s1.pdf]

Supplementary Information - Reduction of nutrients, microbes, and personal care products in domestic wastewater by a benchtop electrocoagulation unit

E.M. Symonds[a,*], M.M. Cook[a], S.M. McQuaig[b], R.M. Ulrich[a], R.O. Schenck[a], J.O. Lukasik[c], E.S. Van Vleet[a], M. Breitbart[a]

[a] *University of South Florida, College of Marine Science, 140 7th Avenue South, St. Petersburg, Florida, USA*
[b] *St. Petersburg College, 2465 Drew Street, Clearwater, Florida, USA*
[c] *BCS Laboratories, Inc., 4609-A NW 6th Street, Gainesville, Florida, USA*

* *Corresponding author. esymonds@mail.usf.edu. +1 (727) 553-3357*

Methods

Bacterial cultures and spores

Enterococcus faecalis ATCC-29212™ and *Escherichia coli* strain C600, grown separately overnight in Nutrient Broth (Difco™, Franklin Lakes, NJ, USA), were added to domestic wastewater samples to yield an approximate final concentration of 10^6 colony forming units (cfu)/ml prior to EC treatment. *Bacillus subtilis* spores were also added to the aforementioned samples to reach an approximate final concentration 10^5 cfu/ml. The *B.subtilis* spores were cultivated one week prior to the experiment from a pure broth culture of *B.subtilis* ATCC-19659™ per Standard E2197-11 of ASTM International [1]. Briefly, the *B.subtilis* culture was inoculated into five liters of 1:10 diluted Columbia Broth (Neogen Inc., Lansing, MI, USA) supplemented with 0.1 mM $MnSO_4 \cdot 4H_2O$. The culture was examined microscopically to verify that >95% of the cells were in the endospore state. The suspension was then heated to 75°C while agitating for 15 min in a water bath to kill vegetative cells and immediately cooled in an ice bath. Spores were enumerated by diluting in phosphate-buffered water (Weber Scientific, Hamilton, NJ, USA), spread plating onto Tryptic Soy Agar (TSA, Neogen, MI) and incubating at 36.5°C for 24 hours.

Viral cultures

Domestic wastewater samples were augmented with pure cultures of human JC polyomavirus (HPyV) ATCC-VR-1583™ and pepper mild mottle virus (PMMoV; provided by Scott Adkins, USDA) to reach an approximate final concentration of 10^6 virus qPCR targets/ml and 10^8 virus qPCR targets/ml, respectively. A purified culture of male-specific (F+) MS2 bacteriophages (ATCC-15597-B1™) was also added to reach approximate final concentrations

of 1.01×10^4 plaque forming units (pfu)/ml. The purified MS2 bacteriophage culture was prepared following the US EPA method 1602 double-agar layer (DAL) protocol for spiking suspensions with MS2 bacteriophages [2]. Minor modifications were made to recover the MS2 bacteriophage culture. Unlike the EPA method 1602 DAL protocol, 10 ml of tryptic soy broth was added to DAL petri plates and plates were incubated at room temperature for 1 hr. The MS2 bacteriophage culture was subsequently recovered using serological pipettes, filtered through a 0.22-µm EMD Millipore Sterivex filter (EMD Millipore, Billerica, MA, USA), quantified to have a concentration of 4.4×10^6 pfu/ml, and stored at 4°C prior to the experiment.

Molecular analysis of human polyomavirus and pepper mild mottle virus

Quantitative PCR (qPCR) and reverse transcription (RT)-qPCR were executed with minor modifications for the analysis of HPyV [3] and PMMoV [4], respectively. Recombinant plasmids, containing either the HPyVs qPCR-target DNA or the PMMoV RT-qPCR-target, were diluted over 5 orders of magnitude to final concentrations ranging from 10^2 to 10^6 targets per µl (e.g. five-point dilution series). Five microliters of each serial dilution were used as target in the HPyV standard curve reactions. HPyV qPCR reactions were prepared by combining 25 µl TaqMan® Environmental Master Mix 2.0 no UNG (Life Technologies, Grand Island, NY, USA), 0.5 µM of each primer (SM2: 5'-AGT CTT TAG GGT CTT CTA CCT TT-3' and P6: 5'-GGT GCC AAC CTA TGG AAC AG-3'), 0.125 µM of the labeled probe (KGJ3: 5'-(FAM)-TCA TCA CTG GCA AAC AT-(MGBNFQ)-3'), 5 µl of template DNA, and nuclease-free water to achieve a final volume of 50 µl [3]. PMMoV qPCR reactions were prepared by combining 12.5 µl Taqman Environmental Mastermix 2.0 no UNG, 0.4 µM of each primer (F: 5'-GAG TGG TTT GAC CTT AAC GTT TGA-3' and R: 5'-TTG TCG GTT GCA ATG CAA GT-3'), 125 nM

probe (5’-(FAM)- CCT ACC GAA GCA AAT G -(TAMRA)-3’), 2 µl of template cDNA, and nuclease-free water to achieve a final volume of 25 µl [4].

For both HPyV and PMMoV analyses, the virus-target concentration of each sample was analyzed in duplicate alongside a 1:10 dilution of sample template (to check for possible PCR inhibition, which was identified when the Cq value of the diluted sample was less than the undiluted sample), all process and extraction controls, no-template controls, and a duplicate standard dilution series with an ABI7500 Real Time PCR system (Life Technologies, Grand Island, NY, USA), according to previously published protocols [3,4]. For HPyV analysis, the qPCR temperature profile was 95°C for 10 min followed by 40 cycles of 95°C for 15 sec, 55°C for 20 sec, and extension at 60°C for 60 sec. The PMMoV qPCR temperature profile was 95°C for 10 min followed by 40 cycles of 95°C for 30 sec, 53°C for 60 sec, and extension at 60°C for 60 sec. All standard curves had regression coefficients >0.97 and qPCR efficiencies between 90 and 110% [5]. Aside from the DNA of one process control showing inhibition for the HPyV assay, no PCR inhibition was observed.

Molecular analysis of Enterococcus

Enterococcus IC-NASBA (Internal control nucleic acid sequence based amplification). An IC-NASBA assay, targeting a 136-bp region of the large subunit ribosomal RNA gene (23s rRNA) of *Enterococcus* spp. related to water quality, was used to determine concentrations of enterococci in augmented wastewater samples before and after EC treatment. The internal control (IC)-RNA was synthesized using *in vitro* transcript generation oligonucleotides (F: 5’-AAT TCT AAT ACG ACT CAC TAT AGG GAG AGA CCC GAA ACC ATG TGA TCT ACC CAT GTC CAG GTT GAA GGT GCG GTA AAA CGC ACT GGA GGA CCG AAC CCA

CGT ACG T-3' and R: 5'-TAT CTC CAA GTT CGT TTG GAA TTT CAT TGT CAC CAT AAG CAG CCA CCC GCA CTT TTC AAC GTA CGT GGG TTC GGT CCT-3'), as previously described [6], and was used to identify amplification inhibition. For each IC-NASBA reaction, the NucliSENS EasyQ® Basic Kit (bio-Mérieux, Durham, NC, USA) was used according to manufacturer's instructions with the following final concentrations: 80 mM KCl (optimized, data not shown), 0.4 mM of each primer (F: 5'-GAC CCG AAA CCA TGT GAT CTA-3' and R: 5'-AAT TCT AAT ACG ACT CAC TAT AGG GAG AAT ATC TCC AAG TTC GTT TGG A-3'), and 0.1 mM of each molecular beacon (Enterococcus: 5'-[6-FAM]-CGA TCG GAT GAG GTG TGG GTA GCG GAC GAT CG-[DABCYL]-3' and IC-RNA: 5'-[6-ROX]-CAT GCG TGG CTG CTT ATG GTG ACA ATC GCA TG-[DABCYL]-3'). Before the addition of 2.5 µl enzyme mix to create a total reaction volume of 10 µl, 2.5 µl of RNA template was added to 5 µl of reagent mixture (primers, beacons, and 1×10^7 IC-RNA copies) and incubated for 2 min at 65°C. Using a NucliSENS EasyQ® analyzer (bio-Mérieux, Durham, NC, USA), NASBA amplification and fluorescence detection occurred at 41°C for 90 min. To quantify enterococci concentrations, *E. faecalis* 29212™ (ATCC, Manassas, VA, USA) whole-cell RNA extracts were serially-diluted from 10^5 to 10^2 cells (4-points) and analyzed in triplicate. The TTP ratio method was used to determine unknown enterococci concentrations from the standard dilutions with a regression coefficient of ≥ 0.95 [6]. No amplification inhibition was observed.

Enterococcus qPCR. An abbreviated version of U.S.EPA Method 1611 was executed, in which only the sections regarding the base TaqMan® (Life Technologies®, Carlsbad, CA) qPCR *Enterococcus* assay were used [7]. Briefly, each TaqMan® qPCR *Enterococcus* reaction had a total volume of 25 µl and contained 1 µM forward primer (5'-GAG AAA TTC CAA ACG AAC TTG-3'), 1 µM reverse primer (5'-CAG TGC TCT ACC TCC ATC ATT-3'), 0.08 µM

TaqMan® probe ([6-FAM]-5'-TGG TTC TCT CCG AAA TAG CTT TAG GGC TA-3'-[TAMRA]), 12.5 µl TaqMan® Universal master mix, 2.5 µl bovine serum albumin (2 mg/ml stock), and 2 µl DNA template. Each sample was analyzed in triplicate with an Applied Biosystems® 7500 Real-Time PCR System (Life Technologies®, Carlsbad, CA, USA) following the previously published thermal profile (50°C for 2 min, 95°C for 10 min, 40x (95°C for 15 sec followed by 60°C for 1 min)) [7]. The standard dilution series, derived from *E. faecalis* 29212™ DNA extracts, contained four points ranging from 10^2 to 10^5 targets per reaction, and was simultaneously analyzed in triplicate alongside samples and no-template controls. The concentrations of *Enterococcus* spp. were calculated based upon the regression analysis of the standard dilution series, which had a regression coefficient of 0.998 and 101% efficiency. All no-template controls were negative. The absence of PCR inhibition was assumed given the lack of inhibition observed during IC-NASBA analyses and the comparable *Enterococcus* spp. concentrations obtained from both methods.

References

1 American Society for Testing and Materials International. ASTM Standard E2197: Standard Quantitative Disk Carrier Test Method for Determining the Bactericidal, Virucidal, Fungicidal, Mycobactericidal and Sporicidal Activities of Liquid Chemical Germicides. (ASTM International, West Conshohocken, PA, 2011).

2 U.S. Environmental Protection Agency. Method 1602: Male-specific (F+) and somatic coliphage in water by single agar layer (SAL) procedure. (Publication No. EPA 821-R-01-029, Office of Research and Development, Washington, D.C., 2001).

3 McQuaig, S. M., Scott, T. M., Lukasik, J. O., Paul, J. H. & Harwood, V. J. Quantification of human polyomaviruses JC virus and BK virus by TaqMan quantitative PCR and comparison to other water quality indicators in water and fecal samples. *Appl Environ Microbiol* 75, 3379-3388 (2009).

4 Rosario, K., Symonds, E. M., Sinigalliano, C., Stewart, J. & Breitbart, M. Pepper mild mottle virus as an indicator of fecal pollution. *Appl Environ Microbiol* 75, 7261-7267 (2009).

5 Bustin, S. A. *et al.* The MIQE guidelines: Minimum information for publication of quantitative real-time PCR experiments. *Clin Chem* 55, 611-622 (2009).

6 Ulrich, R. M. *et al.* Detection and quantification of *Karenia mikimotoi* using real-time nucleic acid sequence-based amplification with internal control RNA (IC-NASBA). *Harmful Algae* 9, 116-122 (2010).

7 U.S. Environmental Protection Agency. Method 1611: Enterococci in water by Taqman® quantitative polymerase chain reaction (qPCR) assay. (Publication No. EPA-821-R-12-008, Office of Water, Washington, DC., 2012).
